# An Electret/Hydrogel-Based Tactile Sensor Boosted by Micro-Patterned and Electrostatic Promoting Methods with Flexibility and Wide-Temperature Tolerance

**DOI:** 10.3390/mi12121462

**Published:** 2021-11-27

**Authors:** Zhensheng Chen, Jiahao Yu, Haozhe Zeng, Zhao Chen, Kai Tao, Jin Wu, Yunjia Li

**Affiliations:** 1 Ministry of Education Key Laboratory of Micro and Nano Systems for Aerospace, Northwestern Polytechnical University, Xi’an 710072, China; 2019201174@mail.nwpu.edu.cn (Z.C.); 1036597329@mail.nwpu.edu.cn (J.Y.); 2018300323@mail.nwpu.edu.cn (H.Z.); chenzhao1999@mail.nwpu.edu.cn (Z.C.); 2 State Key Laboratory of Optoelectronic Materials and Technologies and the Guangdong Province Key Laboratory of Display Material and Technology, School of Electronics and Information Technology, Sun Yat-sen University, Guangzhou 510275, China; 3 School of Electrical Engineering, Xi’an Jiaotong University, Xi’an 710049, China

**Keywords:** flexible electronics, electret/hydrogel-based tactile sensors, pyramidal parented hydrogel, anti-freezing and anti-drying

## Abstract

With the rising demand for wearable, multifunctional, and flexible electronics, plenty of efforts aiming at wearable devices have been devoted to designing sensors with greater efficiency, wide environment tolerance, and good sustainability. Herein, a thin film of double-network ionic hydrogel with a solution replacement treatment method is fabricated, which not only possesses excellent stretchability (>1100%) and good transparency (>80%), but also maintains a wide application temperature range (−10~40 °C). Moreover, the hydrogel membrane further acts as both the flexible electrode and a triboelectric layer, with a larger friction area achieved through a micro-structure pattern method. Combining this with a corona-charged fluorinated ethylene propylene (FEP) film, an electret/hydrogel-based tactile sensor (EHTS) is designed and fabricated. The output performance of the EHTS is effectively boosted by 156.3% through the hybrid of triboelectric and electrostatic effects, which achieves the open-circuit peak voltage of 12.5 V, short-circuit current of 0.5 μA, and considerable power of 4.3 μW respectively, with a mentionable size of 10 mm × 10 mm × 0.9 mm. The EHTS also demonstrates a stable output characteristic within a wide range of temperature tolerance from −10 to approximately 40 °C and can be further integrated into a mask for human breath monitoring, which could provide for a reliable healthcare service during the COVID-19 pandemic. In general, the EHTS shows excellent potential in the fields of healthcare devices and wearable electronics.

## 1. Introduction

The rapid development of wearable and functional electronics has drawn significant attention towards the field of transparent, flexible, and efficient devices that are urgently demanded by human beings [1,2,3,4,5,6,7]. Various devices and applications of mechanical sensors [2,8,9,10], flexible electronic skins [11,12,13,14,15], and wearable devices [16,17,18] have been designed and introduced into our lives, which facilitate the evolution of human science and technology. Various flexible devices have been widely employed and investigated for their merits of easy integration, outstanding biocompatibility, and mechanical characteristics [19,20,21,22,23,24,25,26]. The conventional power supply methods for the devices mentioned above, such as batteries, unavoidably lead to environmental problems. Additionally, the poor flexibility and inconvenient maintainability of traditional devices give rise to severe restrictions for their biophysical applications in wearable electronic device industry. Hence, the capability of harvesting energy from external stimulations, namely self-powered, to drive the devices or generate output signals, is urgently demanded [27,28,29,30,31,32,33,34].

The triboelectric effect has received enormous interest for its effective power generation function and was first proposed by Prof. Zhonglin Wang in 2012 as triboelectric nanogenerators (TENGs) [4]. Subsequently, many absorbing devices have been presented and studied, including TENGs with intense output power density, entirely newly designed structures for ocean energy harvesting, ultra-thin devices for wearable biophysiological sensing, and integration in diverse engineering applications [35,36,37,38,39,40,41,42,43]. Due to the synergistic effect of triboelectrification and electrostatic induction, TENG will generate the output signals continuously according to the external stimulations. When two friction layers with different electron affinities contact and separate with each other, a redistribution of charges happens in the external circuit, which will generate an alternating current responding to the dynamic variations of the stimulations, capable of powering small electronic devices [44].

However, there still remain two significant drawbacks that need to be considered for the self-powered electronics. The first problem is that most flexible TENGs’ performances are limited by environmental temperatures. Traditional electrodes utilized in traditional TENGs are generally metals, which lead to a rigid and opaque device, further restricting their applications seriously [45,46,47,48,49]. Some flexible electrodes, such as liquid metals or elastomeric polymers, are currently adopted by TENGs [11,50,51,52,53], and such materials improve the flexibility and deformable ability a lot but are also constrained by their poor temperature tolerance, resulting in the invalidation phenomenon in extreme environments [54,55,56,57,58,59]. To solve this problem, a double-networked ionic hydrogel with a solvent replacement treatment method is proposed and applied in this work. Due to the excellent stretchability, electrical stability, and wide temperature tolerance of the hydrogel [60], the EHTS device in this paper demonstrates great potential in the field of wearable and flexible electronics under some extreme environments.

The second problem here is that the output performance of most self-powered sensors is primarily constrained by the efficiency of triboelectrification and electrostatic induction, which naturally depends on the materials utilized. To increase the output signals, a micro-pyramid patterned spin coating method is introduced in the hydrogel fabrication process to further enlarge the triboelectric(contacting) area, and thus the output voltage increases significantly. Moreover, a fluorinated ethylene propylene (FEP) film with an effective corona discharging processing method is integrated into the EHTS, which further promotes performance.

**Figure 1:**
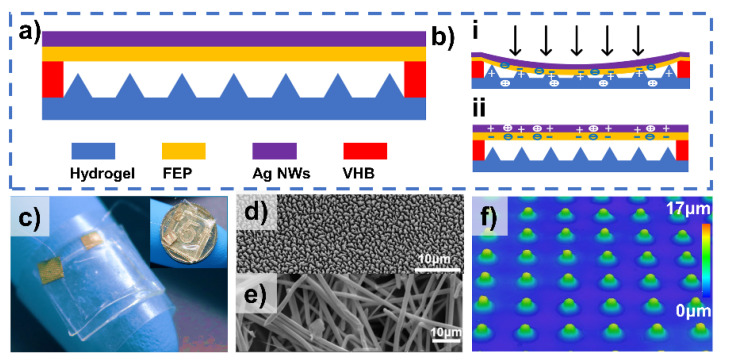
The basic structure of (electret/hydrogel-based tactile sensor EHTS): (**a**) Structure design of the proposed EHTS; (**b**) Schematic diagram of the ETHS at the compressed state (i) and released state (ii); (**c**) Photograph of the fabricated device attached on the fingertip. The inset is the ETHS on a fifty-cent coin; (**d**,**e**) Scanning electron microscopy (SEM) images of the nanostructured fluorinated ethylene propylene (FEP) film surface and the fabricated AgNWs electrode; (**f**) Confocal Laser Scanning Microscope (CLSM) image of the micro-pyramidal structures on the hydrogel.

In this article, we proposed an electret/hydrogel-based tactile sensor (EHTS) with a pyramidal patterned double-networked (DN) ionic hydrogel membrane and charged FEP electret thin film to realize self-sustained physical detection (Figure 1a). As is implemented in our previous work [2], conductive silver nanowires (AgNWs) synthesized with polyethylene terephthalate (PET) film were adopted as the flexible substrate for the AgNWs electrode. The fabricated EHTS has a basic cavity structure which consists of an FEP and AgNWs film opposite the micro-pyramidal structured DN ionic hydrogel film. During the simple operating mode of pressing and releasing, both triboelectric and electrostatic effects occured to generate the external currents between the top AgNWs and the bottom hydrogel electrodes (Figure 1b(i,ii)). Due to the high transparency and deformability of hydrogel and FEP and AgNWs layers, EHTS demonstrates excellent visibility and flexibility (As is demonstrated in Figure 1c, ETHS could be easily adhered to the fingertip. Inset image shows the size of the EHTS on top of a fifty-cent coin). The scanning electron microscope (SEM) images of the nanostructured FEP film (Figure 1d) and AgNWs electrode (Figure 1e) show the roughness surface topography, which boosts the ability to capture more charges to improve the performance of EHTS. Besides, to further increase the contact area to enhance the output performance of EHTS, micro-pyramidal patterned hydrogel (confocal laser scanning microscope image of Figure 1f) was employed to promote the triboelectric effect of the device. 

## 2. Materials, Characteristics and Fabrication Methods

**Figure 2:**
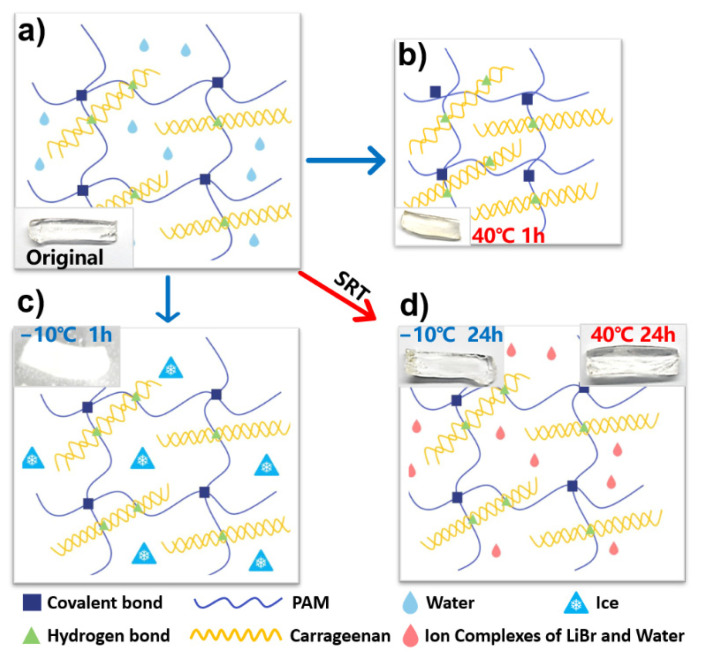
(**a**) Schematic illustration of the double-networked (DN) hydrogel without solvent replacement treatment (SRT) at original state. Inset is the photograph of the original hydrogel; (**b**,**c**) Dehydration and freeze state of the original DN hydrogel when stored at 40 °C and −10 °C for 1 h; (**d**) Anti-freezing and anti-drying properties of DN hydrogel after SRT.

Traditional DN hydrogel is often supposed to be a promising material for flexible and transparent electronics due to its ultra-high deformability, outstanding transparency, excellent mechanical strength, and ionic conductivity. However, the above properties of DN hydrogel can be seriously affected by the external temperature, which is mainly due to the large water content of the hydrogel (>80% usually). Thus, the performance of hydrogel-based electronics is often constrained by their low-temperature tolerance, which leads to severe limitations of their practical value. Herein, a novel solvent replacement treatment (SRT) based on a saturated solution of LiBr is utilized in the hydrogel fabrication process to improve the temperature tolerance. To investigate the wide-temperature tolerance of the SRT hydrogel, comparison tests between the pristine hydrogel and the hydrogel with SRT are presented. A carrageenan/polyacrylamide (PAM) DN hydrogel is utilized in this work as the pristine hydrogel by a simple thermal polymerization method. The PAM and carrageenan can be naturally cross-linked via covalent bonds and particle bonds (As is schematically demonstrated in Figure 2a). The manufactured DN hydrogel is cut into five samples with the same size of 20 mm × 5 mm × 5 mm to investigate the environmental tolerance properties between the pristine hydrogel and the hydrogel with SRT. Two samples are immersed in the 50 wt% LiBr solution (saturated solution at room temperature) to fabricate the hydrogel samples with SRT while the others stay untreated. The pristine DN hydrogel sample shows excellent flexibility and transparency as shown in Figure 2a. However, free water molecules in the pristine hydrogel remain unbonded, which will freeze or evaporate naturally due to the temperature change in the external environment. After being kept in the oven (set for 40 °C) for 1 h, the pristine hydrogel shrunk severely due to the rapid evaporation of water (shown in Figure 2b), which results in the severe dehydration and significant rigidity of the test sample (inset in Figure 2b). On the contrary, the counterpart with SRT remains unchanged under the same condition due to the formation of ion complexes between the LiBr and water molecules (right inset of Figure 2d), which leads to the strong ionic hydration effect of the hydrogel with SRT (Figure 2d). For the extreme cold condition, Figure 2c schematically illustrates the phase transformation of the free water molecules in the pristine hydrogel. After being stored at −10 °C in a refrigerator for 1 h, a test sample of pristine hydrogel thoroughly froze (inset in Figure 2c). Conversely, owing to the low freezing point of LiBr solution, no icing phenomenon is found in the DN ionic hydrogel sample with SRT at the same experimental conditions.

**Figure 3:**
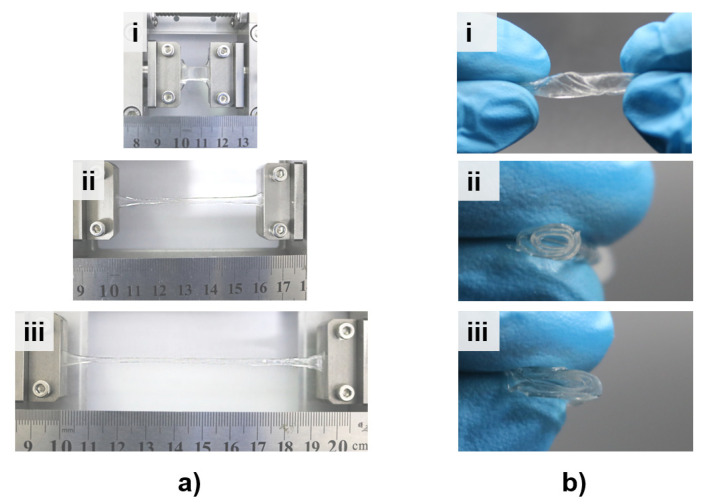
(**a**) Elongation test of the fabricated DN ionic hydrogel at 0% (i), 750% (ii) and 1180% (iii); (**b**) Photographs of the DN ionic hydrogel membrane at twisting (i), rolling (ii) and folding (iii) state.

**Figure 4:**
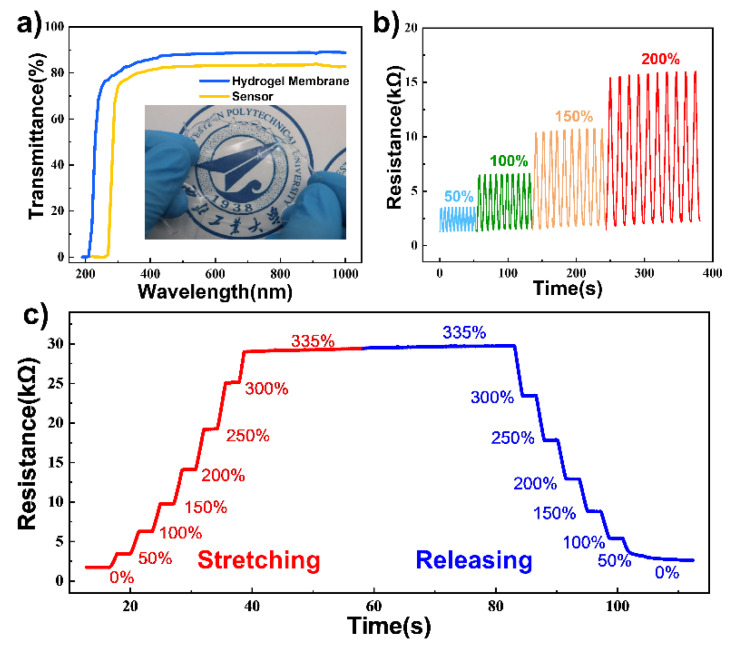
(**a**) Transmittances of the fabricated DN ionic hydrogel membrane and the EHTS; (**b**) Resistance variation of the hydrogel under different cyclic stretching states; (**c**) Real-time response of the hydrogel resistance when stretched to 335% and then released.

Due to the strongly interconnected networks of carrageenan and PAM chains as well as the covalent bonds and hydrogen bonds in the DN ionic hydrogel (as is demonstrated in Figure 2d), excellent stretchability and flexibility can be achieved. The stretch test of the fabricated DN ionic hydrogel is implemented in Figure 3a. The original hydrogel sample (cut to 7.5 mm × 4 mm × 3 mm) can be easily stretched over 1180% in a mechanical tensile platform (Figure 3a(i–iii)), demonstrating the promising ductility of the DN ionic hydrogel. By a simple spin coating method, DN ionic hydrogel with SRT (hereinafter referred to as SRT hydrogel) achieves ultra-thin thickness within 200 μm. Therefore, the fabricated SRT hydrogel membrane can be easily twisted, rolled, and folded (Figure 3b(i–iii)), which contributes a lot to the excellent flexibility as well as the considerable transparency of the fabricated EHTS. As the transmittance testing results shown in Figure 4a, the SRT hydrogel membrane and EHTS remain around 90% and 80% luminousness in the range of 400 nm~800 nm (visible region), respectively. A logo beneath the SRT hydrogel membrane can be observed clearly (as the inset of Figure 4a), which could bring tremendous application potential for EHTS in wearable electronics. 

Additional experiments have been carried out to evaluate the resistance performance while stretching the SRT hydrogel (Figure 4b). The dynamic resistance variation shows the excellent electrical stability of the SRT hydrogel sample in the cyclic test (10 cycles for 50%, 100%, 150%, and 200% strain state, respectively). Additionally, the resistance of SRT hydrogel is positively correlated with the stretching state. As the tensile strain increases from 0% to 335% sequentially, resistance changes accordingly. The same trend can also be found in the releasing process (Figure 4c). Therefore, we chose SRT hydrogel as the flexible electrode as well as the triboelectric layer in EHTS.

**Figure 5:**
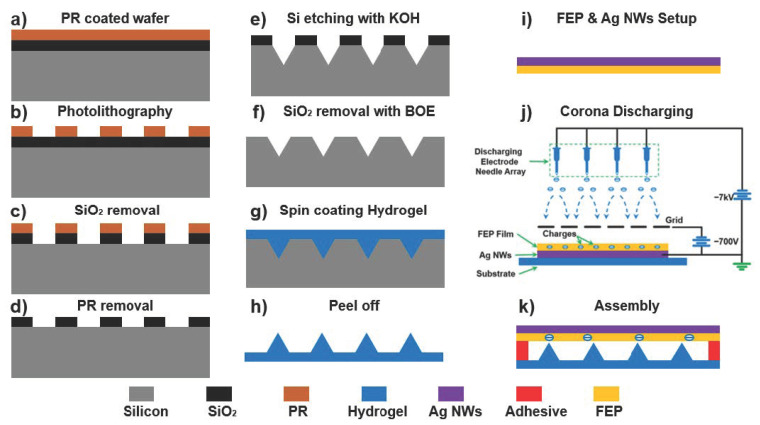
Fabrication of the micro-pyramidal patterned hydrogel membrane and EHTS. (**a**) The thermally oxidized Si wafer was spin-coated with Shipley1805 photoresist (PR); (**b**) PR was partial removed through photolithography; (**c**) The SiO_2_ was etched in BOE plasma; (**d**) PR was completely removed; (**e**) Si etching with KOH to form the inverted pyramids array; (**f**) SiO_2_ layer was removed with BOE thoroughly; (**g**,**h**) The micro-pyramidal patterned hydrogel film was spin-coated and peeled off; (**i**) FEP electret film and AgNWs electrode are bonded together with ethoxylate resin; (**j**) Corona discharging method is adopted to implant charge into FEP film; (**k**) EHTS was fabricated by assembled micro-pyramidal patterned hydrogel film and charged FEP & AgNWs together.

To fabricate EHTS, a production process has been put forward, including a series methods of Si wafer etching, spin coating, peeling off, FEP discharging, and final assembly (as shown in Figure 5). The Si wafer (100) was oxidized in a furnace to grow a SiO_2_ layer of 300 nm thickness on the substrate surface. The thermally oxidized Si wafer is spin-coated by the Shipley1805 (MicroChem, Round Rock, TX, USA) photoresist (PR) (Figure 5a). As is demonstrated in Figure 5b, the Cr-coated hard photomask was utilized to fabricate the blank square array on PR layer (by SUSS MJB4 UV400, SUSS MicroTec company, Garching, Germany). After the exposure process of PR, the wafer was immersed in the buffered oxide etch (BOE, NH4F/HF = 7:1, *v*/*v*, Transene Company, MA, USA) for 4 min to remove the SiO_2_ under the square array (Figure 5c). Then, the PR was removed by sonication in acetone (Figure 5d). The anisotropy of the wet etching by KOH etching solution (30% KOH in H_2_O/isopropanol (4:1 *v*/*v*)) was carried out for 2 mins, ensuring to format the inverted pyramidal structures (with the) on the Si surface (Figure 5e). To complete the fabrication of the Si mold, the SiO_2_ layer was removed through BOE solution treatment (Figure 5f). The micro-pyramid patterned DN hydrogel membrane can be achieved by spin coating the pre-mixed hydrogel solution at the speed of 800 rpm for 15 s (Figure 5g). After the formation, the DN hydrogel membrane is peeled off, followed with the SRT to acquire the SRT hydrogel layer (Figure 5h). As shown in Figure 5i, the AgNWs/FEP composite is prepared as the triboelectric layer and flexible electrode by a typical process method described in our previous research. The fabricated AgNWs/FEP film is charged by the corona discharging system (Figure 5j). Finally, the formed micro-patterned SRT hydrogel is assembled with the charged AgNWs/FEP film with double-sided tape to ensure the charged FEP film is opposite the micro-patterned hydrogel surface (Figure 5k). At last, EHTS is completely fabricated by connecting the hydrogel and AgNWs electrodes together.

## 3. Working Principles

**Figure 6:**
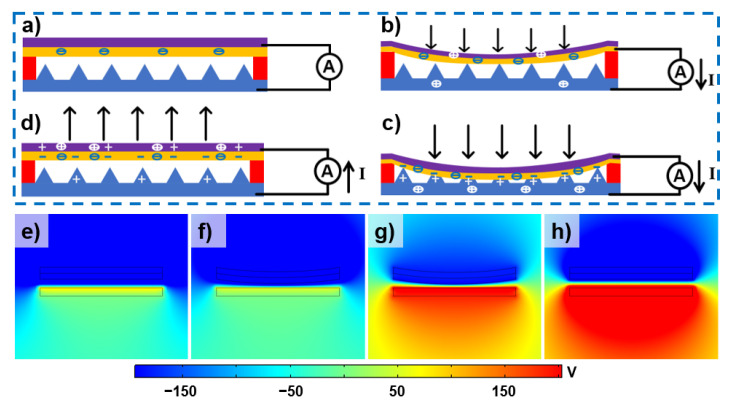
Working principle of the EHTS: Original (**a**), Compressing (**b**), Compressed (**c**) and recovered (**d**) state; Electric field variation of EHTS during the compress-recovery cycle (**e**–**h**).

Figure 6 shows the charge circulation and the electric field variations of the fabricated EHTS in the compress–release cycle. Figure 6a demonstrates the initial state without any stimulation, when charged FEP/AgNWs film and hydrogel layer are opposite with, and separate from each other. After the external force is applied on EHTS, the gap distance between two plates decreases and electrostatic induction happens naturally. The implanted electrons in FEP film induce the positive charges transfer through the external circuit to generate the current (Figure 6b). As the deformation intensifies, the triboelectrification effect occurs when the FEP film contacts the hydrogel surface, which will further increase the output current (Figure 6c). After the compressing force is removed, EHTS will recover to its original state and drive the charges flow back to the balanced state again (Figure 6d). During this cycle, both electret-based electrostatic and triboelectric effects work together to magnify the output performance of EHTS. Moreover, due to the deformation of the micro-pyramids on the hydrogel surface, the contact area is increased, which leads to a stronger triboelectrification effect. Numerical simulations of EHTS have also been calculated by Comsol Multiphysics software (Version 5.5, COMSOL, Inc., Stockholm, Sweden) to analyze the potential variations. Figure 6e shows the initial potential distribution of EHTS at the original state. The upper FEP film has already been corona charged, which provided the strong negative potential. As the applied force increases, the distance between FEP film and hydrogel layer becomes closer due to the compressing deformation of the FEP & Ag NWs layers, which causes electrostatic induction and the variation of the potential (Figure 6f). Eventually, with the increase of the applied pressure, FEP film fully contacted the bottom hydrogel. The different electron affinities between the hydrogel electrode and FEP film lead to the triboelectric effect in the contact interface. The potential difference between the two friction layers has been dramatically increased (Figure 6g). Thus, the output performance of EHTS has been boosted significantly by the combination promoting efforts of the triboelectric and electrostatic effects. As a result, potential distribution varies accordingly when two contacted layers separate to their original states (Figure 6h).

## 4. Results

**Figure 7:**
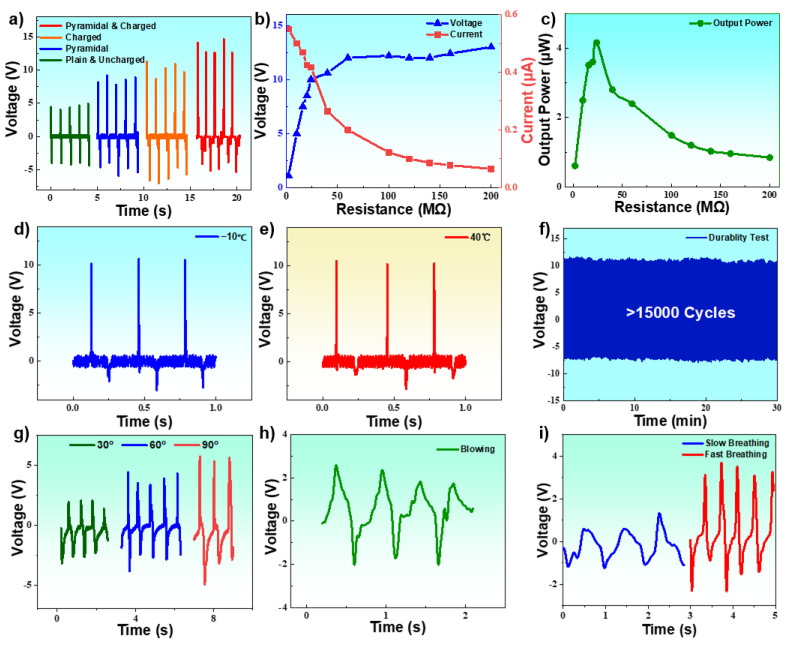
(**a**) Comparison of the output performance between plain & uncharged, pyramidal hydrogel & non-charged FEP, plain hydrogel & charged FEP and pyramidal hydrogel & charged FEP; (**b**) Output voltages and currents of EHTS at different load resistances by shaker tapping; (**c**) The optimization of output power with different load resistances; Output performance of EHTS at −10 °C (**d**), 40 °C (**e**); (**f**) Long-time durability test over 15,000 cycles; (**g**) Output signals of EHTS when be bent for 30°, 60° and 90°; (**h**) Dynamic output of the EHTS attached to a mask when man blowing; (**i**) Output performances of EHTS attached into a mask to detect the dynamic breath of a person.

The enhanced effects of the output performance obtained by micro-pyramidal patterned method and charged FEP electret have been further investigated. Four kinds of EHTSs are fabricated by different process methods, such as the EHTS with plain hydrogel and non-charged FEP, the device with micro-pyramidal patterned hydrogel but non-charged FEP, the plain hydrogel with charged FEP, and the EHTS with both microstructures and charged FEP. The output voltages of the three EHTSs are measured at the same stimulation conditions by a vibration platform at 1 Hz. As we can obviously conclude in Figure 7a, the 1st, 2nd, 3rd, and 4th EHTS outputs the signals with the average peak voltage of 4.8 V, 8.6 V, 9.8 V, and 12.3 V, respectively. Therefore, the enhancement rates of the micro-pyramidal patterned method and charged FEP film are about 79% and 104%. With the combined effect of microstructures and FEP electret, EHTS generates a considerable peak open-circuit voltage of 12.5 V and an impressive short-circuit current of 0.54 μA (Figure 7b). The maximum output voltage of the experiments result is approximately only 1/10 of the simulation one. The output voltage is related to the charge density of the electret according to the relative research implemented by X. Ye, et al. [31]. During negative corona charging, a lot of positive charges are injected into the back-side of the hydrogel surface, which indicate the double sides surface potential are pretty close. Therefore, difference between the front and back surface charge densities is much smaller than the charge density of the front side, which is identified as the main reason for the much lower result. As is shown in Figure 7c, the maximum instantaneous output power of EHTS achieves 4.3 μW with an optimum load resistance of 24 MΩ. What needs to be emphasized is that all the above output performances are generated by the EHTS with the small size of 10 mm × 10 mm × 0.9 mm, which is sufficient to be integrated in wearable electronics and sensors with a strict dimension limitation.

To explore the influence of the external temperature variations on the output performance of the EHTS, comparative experiments have been designed to test the voltages of the EHTS under −10 °C and 40 °C. The results are shown in Figure 7d,e. With a simple finger compressing stimulation, EHTS can generate the output signals around a peak voltage of 10 V with no noticeable variations among a wide temperature range from −10 °C to 40 °C. The excellent output stabilities under the extreme temperature environment can be mainly attributed to the extraordinary anti-freezing property of the flexible SRT hydrogel, which will further broaden the applicability of the self-powered wearable electronics. In addition, a long-time durability test is also investigated, and EHTS has been tested using a mechanical shaker at a constant frequency of 10 Hz continuously. Hence, EHTS shows excellent durability, and the output voltage remains unchanged after 15,000 cycles. No apparent structural damage occurred during this period. The fabricated EHTS demonstrates great flexibility as well as considerable output performance during bending. As is demonstrated in Figure 7g, EHTS can generate peak voltages around 2.3, 2.9, and 5 V, respectively, when being bent at 30°, 60°, and 90°.

Finally, the fabricated EHTS is assembled into the two filter layers of a mask to monitor dynamic breaths. Although the stimulation strength of man’s breath is too weak to activate traditional TENGs, a clear signal with the peak voltage around 2 V can still be acquired by integrating the EHTS between the two filter layers of a mask (Figure 7h). EHTS is further attached in a mask to monitor dynamic breath frequency. Figure 7i significantly demonstrates different peak voltages and frequencies when the tester breaths slow (1 Hz) and fast (~2.5 Hz), which offers a considerable method for healthcare monitoring during the COVID-19 pandemic.

## 5. Conclusions

In this paper, an electret/hydrogel-based tactile sensor combined with the pyramidal patterned DN SRT ionic hydrogel membrane and FEP electret thin film is proposed and researched in detail. To enhance the temperature tolerance of the DN ionic hydrogel, a LiBr solvent replacement treatment is introduced to fabricate the SRT hydrogel. Due to this improvement method’s low freezing point and strong hydration effect, SRT hydrogel demonstrates great anti-freezing and anti-drying properties (from −10 to 40 °C), which are both critical capabilities for wearable electronics and devices. The mechanical stretchability and electrical stability of the SRT hydrogel with double networked structures are further investigated, while ultra-elongation (>1100%) of the hydrogel sample is obtained along with stable resistance responses while stretching. Multi-layered encapsulation and the spin-coating method were utilized to produce the hydrogel membrane and the whole sensors, ensuring the flexibility (including twisting, folding, and rolling for hydrogel membrane), ultra-thin thickness (<1 mm), and excellent transmittance (around 80% in the visible spectral range) of the EHTS. Moreover, the microstructure patterned SRT hydrogel is fabricated with a typical MEMS process, significantly enhancing the triboelectric output performance by 87.5%. Not only is the triboelectrification strengthened, but the electrostatic induction is also boosted. With a customized corona discharging platform, the FEP film is implanted with the negative charges, which increases EHTS output performance efficiency by 77%. The output power of 4.3 μW is obtained by the EHTS with the small size of only 10 mm × 10 mm × 0.9 mm, and the wide temperature tolerance (−10 to 40 °C) of EHTS is achieved simultaneously.

In general, the hydrogel utilized in this research demonstrates great flexibility, outstanding transparency, excellent stretchability, and anti-freezing/drying properties, which provides an ideal electrode and triboelectric friction layer solution for self-powered wearable electronics. Combined with the triboelectrification and electrostatic induction effects, the self-powered tactile sensor proposed in this paper demonstrates larger output performance and considerable sensitivity, and can even be integrated into a mask to monitor the breath during the COVID-19 pandemic. Therefore, EHTS shows great potential in the field of healthcare devices and wearable electronics.

## Data Availability

The data that support the findings of this study are available from the first author upon reasonable request.
